# Mobile addiction treatment and harm reduction services as tools to address health inequities: a community case study of the Brockton Neighborhood Health Center mobile unit

**DOI:** 10.3389/fpubh.2024.1407522

**Published:** 2024-06-18

**Authors:** Allyson Pinkhover, Kelly Celata, Trevor Baker, Avik Chatterjee, Karsten Lunze

**Affiliations:** ^1^Brockton Neighborhood Health Center, Brockton, MA, United States; ^2^Johns Hopkins Bloomberg School of Public Health, Johns Hopkins University, Baltimore, MD, United States; ^3^Boston Medical Center, Boston, MA, United States; ^4^Boston University Chobanian and Avedisian School of Medicine, Boston, MA, United States

**Keywords:** overdose, harm reduction, substance use, mobile health, health equity, opioid use disorder, addiction treatment, outreach

## Abstract

Opioid overdose deaths continue to increase in the US. Recent data show disproportionately high and increasing overdose death rates among Black, Latine, and Indigenous individuals, and people experiencing homelessness. Medications for opioid use disorder (MOUD) can be lifesaving; however, only a fraction of eligible individuals receive them. Our goal was to describe our experience promoting equitable MOUD access using a mobile delivery model. We implemented a mobile MOUD unit aiming to improve equitable access in Brockton, a racially diverse, medium-sized city in Massachusetts. Brockton has a relatively high opioid overdose death rate with increasingly disproportionate death rates among Black residents. Brockton Neighborhood Health Center (BNHC), a community health center, provides brick-and-mortar MOUD access. Through the *Communities That* HEAL intervention as part of the *HEALing Communities Study* (HCS), Brockton convened a community coalition with the aim of selecting evidence-based practices to decrease overdose deaths. BNHC leadership and coalition members recognized that traditional brick-and-mortar treatment locations were inaccessible to marginalized populations, and that a mobile program could increase MOUD access. In September 2021, with support from the HCS coalition, BNHC launched its mobile initiative – Community Care-in-Reach® – to bring low-threshold buprenorphine, harm reduction, and preventive care to high-risk populations. During implementation, the team encountered several challenges including: securing local buy-in; navigating a complex licensure process; maintaining operations throughout the COVID-19 pandemic; and finally, planning for sustainability. In two years of operation, the mobile team cared for 297 unique patients during 1,286 total visits. More than one-third (36%) of patients received buprenorphine prescriptions. In contrast to BNHC’s brick-and-mortar clinics, patients with OUD seen on the mobile unit were more representative of historically marginalized racial and ethnic groups, and people experiencing homelessness, evidencing improved, equitable addiction care access for these historically disadvantaged populations. Offering varied services on the mobile unit, such as wound care, syringe and safer smoking supplies, naloxone, and other basic medical care, was a key engagement strategy. This on-demand mobile model helped redress systemic disadvantages in access to addiction treatment and harm reduction services, reaching diverse individuals to offer lifesaving MOUD at a time of inequitable increases in overdose deaths.

## 1 Introduction

Overdose deaths have disproportionately affected racial and ethnic minoritized groups in recent years. A study examining overdose death rates in the U.S. by age, sex, race, and ethnicity during the COVID-19 pandemic demonstrated that in 2021, non-Latine Black (hereafter referred to as Black) men accounted for the highest rate of overall overdose deaths and overdoses, involving fentanyl at rates of 61.2 deaths per 100,000 population and 43.3 per 100,000, respectively (1). Comparable trends took place in Massachusetts the same year. Overdoses among Black individuals increased 70% between 2019 and 2020, and again by 42% between 2021 and 2022. At 143.8 per 1,000,000 in 2022, overdose rates for American Indian/Alaska Native individuals (hereafter referred to as Indigenous) are 433% of the rate for the general population (2). Similarly, opioid-related overdoses increased among Latine individuals (hereafter referred to as Latine) from 35.4 per 100,000 in 2020 to 39.1 and 45.5 per 100,000 in 2021 and 2022, respectively (2). Meanwhile, a decrease in opioid-related overdose death rates was observed among White individuals (36.5 per 100,000 in 2021 versus 33.3 per 100,000 in 2022) (2). Of note, the authors acknowledge that bodies of literature employ widely varied descriptors of racial and ethnic groups. Hereafter, formatting of descriptors will align with preferred terminology from the American Medical Association’s *Inclusive Language for Reporting Demographic and Clinical Characteristics* (3). When referring collectively to individuals with Black, Latine, and/or Indigenous racial and ethnic identities, the terms marginalized, historically marginalized or oppressed, or underrepresented racial or ethnic groups may be used (3).

Through a social justice and Right to Health lens, it is evident that structural racism, racialized discriminatory drug policy, and subsequent systemic disadvantages result in disparities in accessibility, availability, acceptability, and quality (AAAQ) of addiction treatment and its outcomes for minoritized racial and ethnic groups (4, 5). Buprenorphine is an evidence-based medication for opioid use disorder (MOUD) that is widely considered to be a first line form of treatment (6). Inequities in AAAQ to this treatment modality for Black, Indigenous and Latine groups are well-documented. In recent years, availability of buprenorphine has increased overall in the United States, however the greatest increases in numbers of buprenorphine providers have occurred in zip codes with highest percentages of White residents (7). A study at the neighborhood level has shown that predominantly Black and Latine communities have less physical availability of buprenorphine (8).

Patients from minoritized racial backgrounds experience delays in accessing addiction treatment (9). When patients from minoritized backgrounds are enrolled in treatment, treatment programs are unsuccessful at retaining them (10–12). Similarly, patients from minoritized backgrounds are at a greater risk for discontinuation of treatment once enrolled than their White counterparts (13). Delays in accessing medical care, and early termination from treatment are attributed to racial discrimination in healthcare settings (14, 15). These factors cumulatively contribute to unequal access to addiction treatment and differential treatment outcomes for racial and ethnic minority communities.

### 1.1 Disparities in MOUD treatment among Black, Latine, and Indigenous peoples

It is well documented that individuals who identify as Black are less likely to receive MOUD treatment, especially buprenorphine (16). A study using data of the *National Ambulatory Medical Care Survey* and *National Hospital Ambulatory Medical Care Survey* from 2004 to 2015 found that Black patients have a statistically significantly lower odds of receiving buprenorphine treatment from ambulatory care facilities than White patients of the same facilities (17). A review of a national commercial insurance database exposed that Black adolescents and young adults are less likely than their White counterparts to receive buprenorphine treatment for opioid use disorder (14). An assessment of Medicaid recipients found that Black individuals were 42% less likely to receive buprenorphine than White patients (18).

Disparities in MOUD access also exist among people with Latine identities. Mirroring results found among their Black counterparts, Latine adolescents and young adults are less likely to receive treatment with buprenorphine than White patients (14). A 2022 analyses of sociodemographic differences in buprenorphine treatment among Medicaid recipients were less likely to receive effective dosing and treatment duration with buprenorphine (19). A similar study found that Latine individuals were 22% less likely to receive buprenorphine than their White counterparts (18).

Like observations among Black and Latine individuals, unequal receipt of MOUD treatment occurs among Indigenous peoples. Medicaid recipients of Indigenous identities had 12% lower odds of receiving buprenorphine treatment than those identified as White (18). A 2021 study found slightly lower retention in buprenorphine care 90 days after initiation among Indigenous patients and identified a dearth of literature focused on MOUD access for this patient population (12). Geospatial analyses of access to buprenorphine treatment has also revealed that census block groups with majority Indigenous populations experience the longest median road distance to a buprenorphine provider among any racial or ethnic group majority census block population (16.5 miles versus 2.1 miles general population) (20).

Root causes of these disparities in receipt of buprenorphine treatment among marginalized individuals can be proximally traced to interpersonal and organizational discrimination, stigma, disproportionately unaddressed social driver of health (SDOH) needs, culturally inappropriate services, and a shortage of providers and locations for buprenorphine treatment, especially those that accept Medicaid plans (16, 21). More distally, discriminatory health care and criminalized drug policies contribute to unequal access in MOUD and addiction treatment in the United States (16, 22, 23).

## 2 Background and rationale

Brockton, Massachusetts is an urban community 20 miles south of Boston. According to the 2020 U.S. Census, 41% of Brockton’s population identifies as Black, 12% are Latine, 0.47% are Indigenous peoples, 32% of residents are foreign-born, and 47% of the population speaks a language other than English in their home (24). Mirroring trends taking place on the state and national levels, Brockton’s historically marginalized populations have been disproportionately impacted by overdose deaths in recent years. Community-level data made available via the National Institutes of Health HEALing Communities study found a 40% increase in overdose deaths among non-Latine Black individuals in Brockton between 2017 and 2021, while overdose deaths for non-Latine White individuals decreased by 8% (25).

The HEALing Communities Study (HCS) is a multi-site study in 67 communities in four states (Kentucky, Massachusetts, New York and Ohio) which uses the Communities that HEAL (CTH) intervention to reduce opioid overdose deaths (26). As part of the Communities That HEAL intervention, (27) developed within the HCS trial, a community coalition was formed in Brockton, comprised of individuals and organizations in Brockton working on the overdose crisis. Health center leadership and coalition members recognized that traditional brick-and-mortar treatment locations were not accessible or acceptable to the entire population, and that a mobile program could increase access to addiction treatment.

In 2020, the HCS coalition in Brockton funded a mobile medical services program operated by Brockton Neighborhood Health Center (BNHC), a Federally Qualified Health Center (FQHC) in Brockton, MA. In September 2021, with support from the HCS coalition, and alongside a partnership with The Kraft Center for Community Health at Massachusetts General Hospital, Brockton Neighborhood Health Center (BNHC) launched its mobile services initiative – Community Care-in-Reach – to bring low-threshold substance use treatment, safer use supplies, and preventive medical care to a patient population at high risk for overdose at sites where overdoses frequently occur.

Equity for marginalized patients, particularly those from oppressed racial and ethnic groups and people experiencing homelessness was a driving force behind program design. BNHC’s mobile services program deconstructed obstacles that contribute to disparities in health outcomes among patients from historically marginalized and oppressed racial and ethnic backgrounds. These challenges included poor accessibility and limited availability of buprenorphine treatment, failure of service providers to engagement patients in treatment, (10) and early discharge from programming for failure to adhere to strict clinic attendance, urine testing, and other requirements (13).

This Community Case Study assesses the equity impact of the Brockton mobile program strategy to increase buprenorphine access. Programs with similar models have demonstrated ability to promote buprenorphine access (28, 29). We hypothesized that a mobile services model that entails accessible MOUD and non-judgmental harm reduction interventions, delivered by a diverse team trained in concepts related to health equity, could effectively promote access for individuals from marginalized racial and ethnic groups to buprenorphine as a first-line treatment and improve engagement in health services for historically oppressed groups, including those experiencing homelessness.

## 3 Essential elements of the intervention

### 3.1 Physical space and logistics

BNHC leaders, architects, and clinical team devised a floor plan for a mobile medical trailer that included features necessary for basic medical care. In addition to developing the internal layout, team members scouted ideal venues for mobile services. Overdose data provided by local law enforcement, feedback from outreach staff and key informants, and proximity to local community partners and pharmacies were considered to determine locations with the greatest demand and feasibility for mobile substance use and medical treatment.

### 3.2 Staffing

The mobile unit is staffed by four team members during each session. A Family Nurse Practitioner serves on the mobile unit to prescribe buprenorphine, pre-exposure prophylaxis for HIV (PrEP), post-exposure prophylaxis (PEP), and provide other basic medical care. An LPN/Phlebotomist is available to draw lab specimens, perform wound care, and assist in administrative tasks such as patient check-in and registration. Peer recovery coaches and community health workers conduct foot outreach near the mobile unit to connect patients to care or inpatient treatment, methadone programs, and other case management services that support general wellness. A program manager oversees the daily operations, including supplies, scheduling, and staffing. For safety reasons, the mobile services unit requires at least three team members to operate and space limitations set the maximum number of staff at five.

BNHC sought to have mobile unit staff composition reflect the diversity of patients being served with the goal of diminishing the possibility of bias. Mobile services staff consisted of nurses, nurse practitioners, community health workers, recovery coaches, and administrators from diverse backgrounds. Leadership recruited staff who spoke Spanish, Cape Verdean Creole, Arabic, Tagalog, and Khmer. BNHC also retains live medical interpreters in Spanish, Portuguese, Cape Verdean Creole, and Haitian Creole. Interpreters at BNHC undergo training related to substance use treatment and harm reduction to ensure comfortable, inclusive care for patients who are best served in languages other than English. Team members have lived experience with addiction, recovery, homelessness, psychiatric conditions, criminal-legal involvement, and military service. Mobile services staff regularly attend training related to health equity, antiracism, and trauma-informed care. The provision of services by a diverse and culturally representative team works to reduce biases, center cultural and linguistic knowledge, and earn trust in the treatment team among patients who have experienced systemic, organizational, and interpersonal discrimination when accessing medical care.

### 3.3 Engaging patients

Accessibility, nonjudgmental communication, and cultural appropriateness are fundamental elements of the mobile services program that promote health equity. The accessible nature of the mobile services program expedites treatment for patients. All appointments are conducted on a walk-in basis, enabling individuals who find it difficult to attend formally scheduled appointments to receive care. The mobile unit parks in locations where patients are known to frequently visit, empowering individuals to access treatment in a comfortable setting. Clinic-based treatment may be postponed by long waitlists, extensive documentation, and biases on behalf of treatment staff. Since the conditions for initiating treatment on the mobile unit are nominal, marginalized patients of the mobile program can receive buprenorphine prescriptions and other medical services without delay.

The immediate availability of a provider improves consistent medication/treatment access by patients, many of whom are members of marginalized racial or ethnic groups, experiencing homelessness, and/or formerly incarcerated. In the brick-and-mortar clinic, patients must call to schedule an appointment, experience wait times, and interact with several staff members before meeting with a medical provider directly. Clinic patients who are tentative to engage in treatment may abandon their efforts entirely because of these obstacles. In the mobile program, a provider is promptly available to prescribe buprenorphine, deliver patient education, and initiate follow up in a setting where patients are known to reside, eradicating these prohibitive factors.

Criteria for participation in mobile services are exceedingly flexible. Patients follow up with the mobile team once a week at a time and place of their preference. Missed appointments, refusal of laboratory testing or urine toxicology testing, or active use typically lead to termination in formal clinic settings. However, none of these factors disqualify patients from accessing care from the mobile program. Since discharge from treatment on the mobile unit is rare, mobile programming promotes accessible care for patients of marginalized backgrounds who are disproportionately affected by early termination from treatment (6).

Patient care in the mobile program centers on non-judgment, enabling providers to earn trust among patients who often/usually experience discrimination within the health care system and have been ostracized and excluded from accessible addiction treatment. The mobile unit team is committed to treating patients in all stages of change, including pre-contemplation and contemplation.

### 3.4 Harm reduction as an engagement tool

In addition to medical care, the mobile unit operates as a Syringe Services Program (SSP), providing safer injection equipment, safer smoking supplies, and naloxone to the community. Anonymous drug checking with fentanyl test strips and Fourier transform infrared spectrometry (FTIR) is also available (30–32). These interventions can reduce the risk of unintentional overdose and infectious diseases and serve to earn trust with the patient population. Patients in active use recognize the mobile program’s provision of services, free of judgment or coercion. When patients eventually require medical care, the rapport necessary to facilitate patient engagement has already been established.

## 4 Methods

To evaluate buprenorphine access, a quantitative assessment of electronic health record (EHR) data from BNHC’s Nextgen and later Epic EHRs was conducted. Encounters resulting in a sublingual buprenorphine prescription were extracted from September 1, 2021 through August 31, 2023 for both the Community Care-in-Reach mobile program and BNHC’s brick-and-mortar office-based addiction treatment program. Prescriptions for extended-release injectable buprenorphine prescribed from the stationary clinic were excluded in this analysis (*n* = 100), which is unavailable in the mobile setting given DEA restrictions on storage and transport of the medication. The resulting report contained all prescription fills in the 2-year timeframe for each patient (e.g., a patient receiving seven refills would appear seven times), which was then deduplicated to obtain a list of unique patients who had received buprenorphine by site. Patients receiving buprenorphine from both locations were represented on both mobile and brick-and-mortar reports. Demographic information for the distinct lists of participants were used to determine aggregate tallies of buprenorphine-receiving patients by race and ethnicity for each clinical site. This allowed for a comparison of percentage of patients receiving buprenorphine by demographic characteristics between the mobile services and brick-and-mortar programs.

## 5 Results

Following 24 months of operation, the mobile services unit has expanded accessibility of MOUD within the Brockton community. From the conception of the program in August 2021 through September 2023, 1,283 medical appointments were conducted for 297 unique individuals. Of these patients, 41% were female, 57% male, 74% were Brockton residents, 7% reported speaking a language other than English, and 29% were experiencing homelessness, of whom half reported staying in a shelter. Two-thirds (66%) identified as White, 25% as Black, 10% as having Latine ethnicity, and 1.0% of Indigenous background. More than half of these visits (59%, *n* = 790) were for the purpose of prescribing buprenorphine (Table 1).

**Table 1:** Demographics of unique patients receiving buprenorphine seen by the Brockton Neighborhood Health Center mobile unit (*n* = 107) and stationary site (*n* = 420), September 1, 2021 through August 31, 2023.

	Mobile unit	Stationary clinic
self-reported racial identity of buprenorphine patients		
Indigenous peoples (American Indian/Alaskan Native)	2 (1.9%)	4 (1.0%)
Asian	0 (0%)	1 (0.2%)
Black	26 (24.3%)	78 (18.6%)
More than one race	2 (1.9%)	13 (3.1%)
Pacific Islander	1 (1.9%)	1 (0.2%)
White	69 (64.5%)	309 (73.6%)
Unknown/refused to report	7 (6.5%)	14 (3.3%)
Total	107 (100%)	420 (100%)
Self-reported Latine racial identity of buprenorphine patients		
Latine	15 (14.0%)	56 (13.3%)
Not Latine	86 (80.4%)	346 (82.4%)
Unknown/refused to report	6 (5.6%)	18 (4.3%)
Total	107 (100%)	420 (100%)

In the two-year operating period, providers on the mobile unit prescribed buprenorphine to 107 unique patients and wrote a total of 695 buprenorphine prescriptions. The average dose per prescription was 17.8 mg per day. Thirty-seven percent of MOUD patients were female and 63% male; approximately 70% were Brockton residents. Those receiving buprenorphine from the mobile unit were as racially diverse (24% Black, 14% Latine, 1.9% Indigenous) as the overall mobile unit patient population and had a similarly high prevalence of homelessness (35%). In contrast, those receiving buprenorphine at the brick-and-mortar clinic were more likely to be White (74%, with 19% Black, 13% Latine, 1.0% Indigenous) than mobile unit buprenorphine patients.

One-third of initial visits to the mobile unit were for wound care. Seventy-four percent of buprenorphine patients presented requesting MOUD access; however, 26% initially presented seeking other services and later returned for buprenorphine treatment (Table 2).

**Table 2:** Reason for encounter at Brockton Neighborhood Health Center mobile unit, August 2021 through September 2023 (*n* = 1,283).

Reason for encounter	Number of encounters (%)
Buprenorphine/MOUD	790 (59%)
Wound care	178 (13%)
HIV/STI testing and/or treatment	96 (7%)
Preventive care/sick visits/misc. follow ups	276 (21%)

The mobile services program has also proven to be an effective tool in reaching individuals experiencing homelessness and individuals reentering the community from incarceration, two groups that are known to be at an elevated risk for overdose (33, 34). Eighty-six (29.0%) of the 297 individual patients the mobile unit served in this period were identified as homeless in the electronic medical record, and 46.1% of homeless patients received buprenorphine prescriptions from the mobile services team. Forty-one patients had been incarcerated in the past 5 years, 65.9% of whom were treated with buprenorphine from the mobile team. It is likely that these data under-represent the prevalence of incarceration and homelessness in the patient population due to limitations of the Electronic Health Record (EHR) in capturing these metrics.

## 6 Discussion

A mobile treatment model for people with OUD that brick-and-mortar treatment fail to engage is feasible. Indeed, similar mobile care models have been implemented successfully to increase access to preventive health and addictions care in diverse settings in the US (28, 35–37). Such models, through geographic and philosophic flexibility, can work to promote health equity and, in part, begin to redress discriminatory practices in the medical system that lead to disparities in MOUD and substance use-related AAAQ measures. At a time when overdose deaths are disproportionately increasing among those identifying as Black, Latine, and Indigenous peoples, it is key that strategies to address the overdose crisis are centered in social justice and a Right to Health framework. The Brockton Community Care-in-Reach® program reached many patients who identified their race or ethnicity from an underrepresented group or reported homelessness, who might otherwise not have MOUD or other forms of medical care accessible to them.

In our experience, harm reduction programs and addiction treatment providers in both mobile and clinic settings should cultivate an atmosphere of acceptance to promote equitable care for patients from marginalized communities. This can be accomplished through thoughtful program design that intentionally eliminates barriers that are reticent of discriminatory policies and practices in the medical system to help promote available, accessible, acceptable, and quality services (23). Nonjudgmental treatment works to foster mutual trust between patients who have experienced current and historical oppression and healthcare providers. Developing culturally appropriate services should be an organizational priority for addiction treatment providers to facilitate patients from historically marginalized racial and ethnic groups to access care. Substance use services should be offered in languages used in the catchment area and delivered in a culturally relevant manner by a multidisciplinary team whose experiences reflect those of the patient population. Staff should participate in regular training relating to antiracism and health equity and be encouraged to use their lived experience to raise up strategies that promote AAAQ. Regularly seeking and incorporating participant feedback into program design is essential.

Establishing a mobile unit involves overcoming many obstacles. Onerous licensing requirements developed with brick-and-mortar sites in mind require reform to encourage mobile programs such as the one highlighted here. Local politics and relationships with other community organizations also determine successful program implementation. To establish a mobile unit, an organization must have strong relationships with existing institutions in the community, which in this case the HCS coalition helped foster.

Clinical takeaways include the importance of wound care and other services because many patients who ultimately received buprenorphine accessed other services first. Providing comprehensive harm reduction supplies including safer injection, sniffing, and smoking kits, is critical to building trust and promoting engagement with services. Additionally, some patients attended appointments at both mobile and brick-and-mortar sites. Mobile services added access for patients already engaged at other BNHC sites who might need occasional geographic flexibility. Finally, buprenorphine served a harm reduction purpose. Even patients who might intermittently missed prescription visits would report that having at least some buprenorphine in their system seemed to prevent overdose. Lastly, while patient feedback helped shape clinical offerings, future opportunities include ongoing engagement of patient and community to shape mobile unit operations.

Finances and sustainability are persistent issues for mobile units (28). Seeking out all relevant grant opportunities is vital. Secondly, maximizing billable encounters – and offering a wide variety of billable clinical services, such as wound care – is crucial. Finally, supportive state-level policies are key to sustainability of such interventions. While Medicaid reimbursement policies are a strength in Massachusetts, they may still under-support mobile MOUD and harm reduction programs that offer low-threshold models of care. Finally, telehealth policies that support remote visits are also important since many mobile unit patients require engagement via telehealth at some point in their care. With many patients lacking smartphone access or data plans, reimbursement for audio-only telehealth visits must remain a point of advocacy as COVID-19 policies are repealed across the country. Medicaid expansion, sufficient coverage of OUD-related treatment and services, and telehealth service coverage are vital policies that must be expanded to further support mobile access to lifesaving MOUD at a time of ever-increasing overdose deaths.

In our experience, a mobile unit offering MOUD is feasible and can help promote availability, accessibility, acceptability, and quality of MOUD services to populations that have been disproportionately impacted by oppression, discrimination, and under-resourcing of medical services.

## Data availability statement

The raw data supporting the conclusions of this article will be made available by the authors, without undue reservation.

## Author contributions

AP: Conceptualization, Data curation, Formal analysis, Investigation, Project administration, Writing – original draft, Writing – review & editing, Methodology. KC: Conceptualization, Data curation, Formal analysis, Investigation, Project administration, Writing – original draft, Writing – review & editing. TB: Methodology, Writing – review & editing. AC: Conceptualization, Investigation, Methodology, Supervision, Writing – original draft, Writing – review & editing. KL: Investigation, Methodology, Supervision, Writing – review & editing.

## References

[1] Han B Einstein EB Jones CM Cotto J Compton WM Volkow ND. Racial and ethnic disparities in drug overdose deaths in the US during the COVID-19 pandemic. JAMA Netw Open. (2022) 5:e2232314. doi: 10.1001/jamanetworkopen.2022.32314, PMID: 36125815 PMC9490498

[2] Massachusetts Department of Public Health . Opioid-Related Overdose Deaths, All Intents, MA Residents – Demographic Data Highlights. (2023). Available at: https://www.mass.gov/doc/opioid-related-overdose-deaths-demographics-december-2022/download. Accessed on May 20, 2023.

[3] Frey T . Updated guidance on the reporting of race and ethnicity in medical and science journals. AMWA J. (2023) 38:621–7. doi: 10.55752/amwa.2023.195 34402850

[4] UN Economic and Social Council . General Comment No 14: the right to the highest attainable standard of health (art. 12 of the covenant). UN Comm Econ Soc Cult Rights. (2000) 2000:1–5.

[5] Montel L Ssenyonga N Coleman MP Allemani C. How should implementation of the human right to health be assessed? A scoping review of the public health literature from 2000 to 2021. Int J Equity Health. (2022) 21:139. doi: 10.1186/s12939-022-01742-0, PMID: 36138460 PMC9502920

[6] Substance Abuse and Mental Health Services Administration . Buprenorphine. (2023). Available at: https://www.samhsa.gov/medications-substance-use-disorders/medications-counseling-related-conditions/buprenorphine. Accessed on November 12, 2023.

[7] Schuler MS Dick AW Stein BD. Growing racial/ethnic disparities in buprenorphine distribution in the United States, 2007-2017. Drug Alcohol Depend. (2021) 223:108710. doi: 10.1016/j.drugalcdep.2021.108710, PMID: 33873027 PMC8204632

[8] Goedel WC Shapiro A Cerdá M Tsai JW Hadland SE Marshall BDL. Association of Racial/ethnic segregation with treatment capacity for opioid use disorder in counties in the United States. JAMA Netw Open. (2020) 3:e203711. doi: 10.1001/jamanetworkopen.2020.3711, PMID: 32320038 PMC7177200

[9] Gryczynski J Schwartz RP Salkever DS Mitchell SG Jaffe JH. Patterns in admission delays to outpatient methadone treatment in the United States. J Subst Abus Treat. (2011) 41:431–9. doi: 10.1016/j.jsat.2011.06.005, PMID: 21821378 PMC3205308

[10] Casagrande SS Gary TL Laveist TA Gaskin DJ Cooper LA. Perceived discrimination and adherence to medical care in a racially integrated community. J Gen Intern Med. (2007) 22:389–95. doi: 10.1007/s11606-006-0057-4, PMID: 17356974 PMC1824749

[11] O’Connor AM Cousins G Durand L Barry J Boland F. Retention of patients in opioid substitution treatment: a systematic review. PLoS One. (2020) 15:e0232086. doi: 10.1371/journal.pone.0232086, PMID: 32407321 PMC7224511

[12] Lillie KM Shaw J Jansen KJ Garrison MM. Buprenorphine/naloxone for opioid use disorder among Alaska native and American Indian people. J Addict Med. (2021) 15:297–302. doi: 10.1097/ADM.0000000000000757, PMID: 33074852 PMC10395652

[13] Samples H Williams AR Olfson M Crystal S. Risk factors for discontinuation of buprenorphine treatment for opioid use disorders in a multi-state sample of Medicaid enrollees. J Subst Abus Treat. (2018) 95:9–17. doi: 10.1016/j.jsat.2018.09.001, PMID: 30352671 PMC6354252

[14] Hadland SE Frank Wharam JW Schuster MA Zhang F Samet JH Larochelle MR. Trends in receipt of buprenorphine and naltrexone for opioid use disorder among adolescents and young adults, 2001-2014. JAMA Pediatr. (2017) 171:747–55. doi: 10.1001/jamapediatrics.2017.0745, PMID: 28628701 PMC5649381

[15] Mays VM Jones AL Delany-Brumsey A Coles C Cochran SD. Perceived discrimination in health care and mental health/substance abuse treatment among blacks, Latinos, and whites. Med Care. (2017) 55:173–81. doi: 10.1097/MLR.0000000000000638, PMID: 27753743 PMC5233585

[16] Andraka-Christou B . Addressing racial and ethnic disparities in the use of medications for opioid use disorder. Health Aff. (2021) 40:920–7. doi: 10.1377/hlthaff.2020.02261 34097509

[17] Lagisetty PA Ross R Bohnert A Clay M Maust DT. Buprenorphine treatment divide by race/ethnicity and payment. JAMA Psychiatry. (2019) 76:979–81. doi: 10.1001/jamapsychiatry.2019.0876, PMID: 31066881 PMC6506898

[18] Dunphy CC Zhang K Xu L Guy GP. Racial–ethnic disparities of buprenorphine and Vivitrol receipt in Medicaid. Am J Prev Med. (2022) 63:717–25. doi: 10.1016/j.amepre.2022.05.006, PMID: 35803789 PMC9588682

[19] Landis RK Levin JS Saloner B Gordon AJ Dick AW Sherry TB . Sociodemographic differences in quality of treatment to Medicaid enrollees receiving buprenorphine. Subst Abus. (2022) 43:1057–71. doi: 10.1080/08897077.2022.2060424, PMID: 35442178 PMC9945372

[20] Amiri S Panwala V Amram O. Disparities in access to opioid treatment programs and buprenorphine providers by race and ethnicity in the contiguous U.S. J Subst Use Addict Treat. (2024) 156:209193. doi: 10.1016/j.josat.2023.209193, PMID: 37890620

[21] Hirchak KA Nadeau M Vasquez A Hernandez-Vallant A Smith K Pham C . Centering culture in the treatment of opioid use disorder with American Indian and Alaska native communities: contributions from a National Collaborative Board. Am J Community Psychol. (2023) 71:174. doi: 10.1002/ajcp.12620, PMID: 35997562 PMC9947183

[22] Netherland J Hansen HB. The war on drugs that Wasn't: wasted whiteness, "dirty doctors," and race in media coverage of prescription opioid misuse. Cult Med Psychiatry. (2016) 40:664–86. doi: 10.1007/s11013-016-9496-5, PMID: 27272904 PMC5121004

[23] Jegede O Bellamy C Jordan A. Systemic racism as a determinant of health inequities for people with substance use disorder. JAMA Psychiatry. (2024) 81:225–6. doi: 10.1001/jamapsychiatry.2023.4958, PMID: 38231489

[24] United States Census Bureau . Quick Facts, Brockton, Massachusetts. (2022). Available at: https://www.census.gov/quickfacts/fact/table/brocktoncitymassachusetts/RHI225221. Accessed on October 4, 2022.

[25] Larochelle M Slavova S Root E Feaster D Ward P Selk S. Disparities in opioid overdose death trends by race/ethnicity, 2018-2019. Am J Public Health. (2021) 111:1851–4. doi: 10.2105/AJPH.2021.306431, PMID: 34499540 PMC8561170

[26] Knudsen HK Drainoni ML Gilbert L Huerta TR Oser CB Aldrich AM . Model and approach for assessing implementation context and fidelity in the HEALing communities study. Drug Alcohol Depend. (2020) 217:108330. doi: 10.1016/j.drugalcdep.2020.108330, PMID: 33086156 PMC7531282

[27] HEALing Communities Study Consortium . The HEALing (helping end addiction long-term)^SM^ communities study: protocol for a cluster-randomized trial at the community level to reduce opioid overdose deaths through implementation of an integrated set of evidence-based practices. Drug Alcohol Depend. (2020) 217:108335. 33248391 10.1016/j.drugalcdep.2020.108335 PMC7568493

[28] Regis C Gaeta JM Mackin S Baggett TP Quinlan J Taveras EM. Community Care in Reach: mobilizing harm reduction and addiction treatment Services for Vulnerable Populations. Front Public Heal. (2020) 8:8. doi: 10.3389/fpubh.2020.00501, PMID: 33102413 PMC7545088

[29] Fine DR Weinstock K Plakas I Mackin S Wright J Gaeta JM . Experience with a mobile addiction program among people experiencing homelessness. J Health Care Poor Underserved. (2021) 32:1145–54. doi: 10.1353/HPU.2021.0119, PMID: 34421018

[30] Wallace B van Roode T Pagan F Hore D Pauly B. The potential impacts of community drug checking within the overdose crisis: qualitative study exploring the perspective of prospective service users. BMC Public Health. (2021) 21:1156. doi: 10.1186/s12889-021-11243-4, PMID: 34134698 PMC8207696

[31] Tilhou AS Zaborek J Baltes A Salisbury-Afshar E Malicki J Brown R. Differences in drug use behaviors that impact overdose risk among individuals who do and do not use fentanyl test strips for drug checking. Harm Reduct J. (2023) 20:41. doi: 10.1186/s12954-023-00767-0, PMID: 36978170 PMC10053743

[32] Maghsoudi N Tanguay J Scarfone K Rammohan I Ziegler C Werb D . Drug checking services for people who use drugs: a systematic review. Addiction. (2022) 117:532–44. doi: 10.1111/add.15734, PMID: 34729849 PMC9299873

[33] Yamamoto A Needleman J Gelberg L Kominski G Shoptaw S Tsugawa Y. Association between homelessness and opioid overdose and opioid-related hospital admissions/emergency department visits. Soc Sci Med. (2019) 242:112585. doi: 10.1016/j.socscimed.2019.112585, PMID: 31634808 PMC7023863

[34] Binswanger IA . Opioid use disorder and incarceration - Hope for ensuring the continuity of treatment. N Engl J Med. (2019) 380:1193–5. doi: 10.1056/NEJMp1900069, PMID: 30811904

[35] Leibowitz A Livaditis L Daftary G Pelton-Cairns L Regis C Taveras E. Using mobile clinics to deliver care to difficult-to-reach populations: a COVID-19 practice we should keep. Prev Med Reports. (2021) 24:101551. doi: 10.1016/j.pmedr.2021.101551, PMID: 34522575 PMC8428151

[36] Berk J . A good place to start — low-threshold buprenorphine initiation. N Engl J Med. (2020) 383:701–3. doi: 10.1056/nejmp2017363, PMID: 32813944

[37] Pepin MD Joseph JK Chapman BP McAuliffe C O’Donnell LK Marano RL . A mobile addiction service for community-based overdose prevention. Front Public Heal. (2023) 11:11. doi: 10.3389/fpubh.2023.1154813, PMID: 37538275 PMC10394629

